# Demographic history and genetic differentiation of an endemic and endangered *Ulmus lamellosa* (*Ulmus*)

**DOI:** 10.1186/s12870-020-02723-7

**Published:** 2020-11-17

**Authors:** Huimin Hou, Hang Ye, Zhi Wang, Jiahui Wu, Yue Gao, Wei Han, Dongchen Na, Genlou Sun, Yiling Wang

**Affiliations:** 1 School of Life Science, Shanxi Normal University, Linfen, 041000 P. R. China; 2 Saint Mary’s University, Halifax, Canada

**Keywords:** *Ulmus lamellosa*, *Aat*, ITS, Genetic differentiation, Demographic history

## Abstract

**Background:**

*Ulmus lamellosa* (one of the ancient species of *Ulmus*) is an endemic and endangered plant that has undergone climatic oscillations and geographical changes. The elucidation of its demographic history and genetic differentiation is critical for understanding the evolutionary process and ecological adaption to forests in Northern China.

**Results:**

Polymorphic haplotypes were detected in most populations of *U. lamellosa* via DNA sequencing. All haplotypes were divided into three phylogeographic clades fundamentally corresponding to their geographical distribution, namely THM (Taihang Mountains), YM (Yinshan Mountains), and YSM (Yanshan Mountains) groups. The YSM group, which is regarded as ancestral, possessed higher genetic diversity and significant genetic variability in contrast to the YSM and YM groups. Meanwhile, the divergence time of intraspecies haplotypes occurred during the Miocene-Pliocene, which was associated with major Tertiary geological and/or climatic events. Different degrees of gene exchanges were identified between the three groups. During glaciation, the YSM and THM regions might have served as refugia for *U. lamellosa*. Based on ITS data, range expansion was not expected through evolutionary processes, except for the THM group. A series of mountain uplifts (e.g., Yanshan Mountains and Taihang Mountains) following the Miocene-Pliocene, and subsequently quaternary climatic oscillations in Northern China, further promoted divergence between *U. lamellosa* populations.

**Conclusions:**

Geographical topology and climate change in Northern China played a critical role in establishing the current phylogeographic structural patterns of *U. lamellosa*. These results provide important data and clues that facilitate the demographic study of tree species in Northern China.

**Supplementary Information:**

The online version contains supplementary material available at 10.1186/s12870-020-02723-7.

## Background

Examining how historical and contemporary ecological factors contribute to the demographic history and genetic differentiation of plants is a central question in ecology and evolution. Climatic oscillations have dramatically influenced the demographic history and patterns of genetic diversification in many plant species, particularly during the Pleistocene periods with more frequent glacial–interglacial cycles [1–8]. In Asia, at least four major glaciations are thought to have occurred, which likely affected its flora and fauna, although the glacial advances were not as extensive as that of Europe and North America. Northern China, in particular, experienced severe climatic oscillations throughout the Quaternary, but was never covered by massive ice-sheets [9–11]. The climatic oscillations caused by repeated glaciations shifted species ranges and isolated populations, which could generate distinct population structures, namely, “southern richness” and “northern purity” [12–14].

Meanwhile, the potential role of geology in facilitating the population divergence and species diversification of plants has been revealed [9, 11, 15, 16]. In China, the geographical and physiognomy complexity caused by the uplift of mountains [17, 18] often provided dispersal corridors for the connection, gene flow, and range expansions/contractions of fragmented populations [19, 20]. Mountainous areas might serve as shelters for the range shifts of plants in response to climatic oscillations [21–26]. Some mountains, such as the Taihang, Yanshan, Yinshan, and Qinling Mountains of Northern China, are an important component of the South-North vegetation transect, and crucial biodiversity areas covered with diverse plant biomes, ranging from tropical to cold forests and taiga [27, 28]. These mountains form diverse topographies and wide spectrum environmental conditions, which provided refugia for species during the global scale climatic changes of the Quaternary [29, 30].

Previous researches have revealed different refugia patterns for forest trees in China, such as single refugium, multiple refugia, microrefugia, and cryptic refugia [31–33]. Nevertheless, most studies have focused on the populations or species in the Southern, Southwestern, and Northwestern regions of China [3, 24, 34], with little research being done on the Northern areas. The potential roles of ecological factors (e.g., geologic and climatic oscillations) in promoting the divergence of plant populations in Northern China remain unclear.

*Ulmus lamellosa* (*Ulmus*) is primarily distributed across and endemic in Northern China, which is an important tree in many communities and terrestrial ecosystems. The abundance of decylic acid in the seeds of this species makes it a critical medicinal and light industrial resource [35], and further, the tree trunks have rich ornamental characteristics [36].

Unfortunately, *U. lamellosa* populations are constantly decreasing due to environmental degradation and anthropogenic destruction. Thus, it has been classified as a Class II State-Protected Endangered Plant Species in China [37, 38]. As an ancient and long-lived tree species, *U. lamellosa* might have existed prior to the uplift of Taihang, Yinshan, and Yanshan Mountains, and experienced the climatic oscillations of the Quaternary and even the Tertiary [39]. Consequently, this species was considered to be an ideal species for investigating the influences of geographical topology and climatic dynamics on species divergence in Northern China.

Using chloroplast derived DNA sequences, Liu et al. (2017) preliminarily investigated the molecular phylogeographical structures and biogeographic history of *U. lamellosa*. A significant phylogeographic pattern was found for this tree species, where the intraspecific divergence of all cpDNA haplotypes began to occur in the late Miocene. Most studied *U. lamellosa* populations have not experienced recent expansions. Across the distribution ranges of this species, multiple refuge areas were identified in Northern China [40]. These results provided insights into the phylogeography and demographical history of *U. lamellosa*. However, chloroplast genes have a relatively slower evolutionary ratio than do nuclear genes, are generally of maternal inheritance, and cannot reflect pollen flow in angiosperms [41–43]. Furthermore, the results based on chloroplast genes provide only limited information on the phylogeographic structures and evolutionary history of plants, particularly intra-specifically [25, 44].

For this study, we proposed the hypothesis that *U. lamellosa* had multiple refuges in Northern China, where geological changes and climatic oscillations played important roles in the demographic history of this species. Thus, our main goals were to comparatively investigate the demographic history and genetic differentiation of *U. lamellosa*, analyze the phylogeographic patterns of this boreal tree, identify the refugia patterns during glacial periods, and estimate intraspecies haplotype divergence times during the evolutionary process of this species. These results would shed light not only on the effect mechanisms of climatic oscillations and orogeny of mountains (Taihang, Yanshan and Yinshan Mountains) on the genetic differentiation and distribution patterns of populations, but also offer a theoretical foundation for the investigation of other forest trees across Northern China.

## Methods

### Plant materials

Our field work had been approved by the Chinese government and implemented in compliance with the laws of the People’s Republic of China. Each sampling participant had a permission letter from School of Life Sciences of Shanxi Normal University. By referencing to the phenotypic characteristics of the collected samples, the professor (Su Junxia, Shanxi Normal University) of systematic botany assisted in the classification and identification. Voucher specimens were kept at the Herbarium of School of Life Sciences, Shanxi Normal University (No: 20180703422–20,180,703,436).

**Fig. 1:**
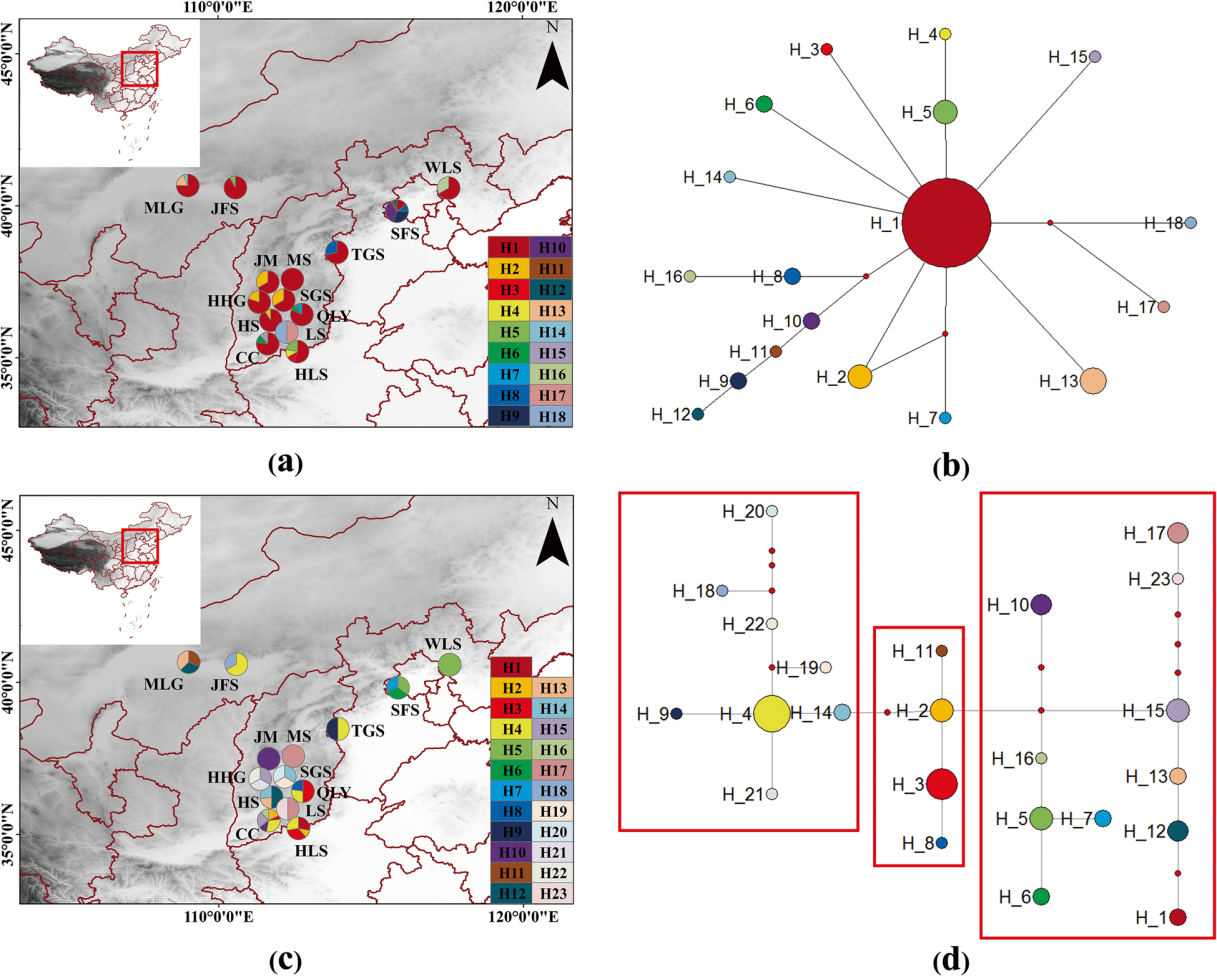
Geographical distribution and the median-joining network of haplotypes. **a** Geographical distribution of 18 ITS haplotypes; **b** The median-joining network of 18 ITS haplotypes; **c** Geographical distribution of 23 *Aat* haplotypes; **d** The median-joining network of 23 *Aat* haplotypes

A total of 232 individuals across fourteen natural populations of *U. lamellosa* were collected from its distribution range in Northern China. According to the filed sampled sites, three main geographical groups were assigned based on mountain boundaries, which were marked as THM (Taihang Mountains), YSM (Yanshan Mountains), and YM (Yinshan Mountains), respectively. Details on the geographical locations of the populations and the number of sampled individuals are presented in Additional file 1: Table S1 and Fig. 1. Individuals within each population need to be kept at least 30 m away from each other to prevent the occurrence of sampling clones. The fresh leaves of each individual were stored in silica gel at room temperature for DNA extraction.

### DNA extraction, amplification, and sequencing

Through Cetyl Trimethylammonium Bromide (CTAB) methods, the total genomic DNA was extracted from fresh leaves [45, 46] followed by the determination of its quality and concentration with the spectrophotometer and agarose gel electrophoresis (0.8%), respectively. The universal primer ITS4 and ITS5 used to amplify of the nuclear ribosomal internal transcribed spacer regions (nrITS: ITS1, 5.8SrDNA and ITS2), and the single-copy gene (*Aat*) [47, 48], were selected for this study due to the high sequence variation within and among *U. lamellosa* populations [40].

The total volume of Polymerase chain reaction (PCR) system was 25 μL, mainly consisting of 2 × MasterMix (12.5 μL, containing 0.2 mM dNTPs, 3 mM MgCl_2_, 1 × PCR buffer and 0.1 U *Taq* DNA polymerase), ddH_2_O (7.5 μL), template DNA (30 ng/μL, 4 μL), and forward and reverse primer (0.5 μL, 0.5 μL). The PCR conditions was performed by using the PTC-200 Peltier Thermal Cycler (MJ Research) as follows: 94 °C predenaturation for 3 min, 34 cycles of denaturation for 30 s, 55 °C annealing for 1 min (for ITS and *Aat*),72 °C elongation for 2 min and a final step at 72C elongation for 10 min.

The PCR products were purified by a Wizard PCR Preps DNA purification system (Promega, Madison, WI, USA) and then sequenced with an ABI Prism 310 DNA sequencer (Applied Bio systems Inc., Foster City, CA, USA) of Shanghai Sangon Biotech Co., Ltd.

The DNA sequences obtained from nrITS and *Aat* were aligned using CLUSTAL X software [49] and then adjusted manually. All indels were encoded into the data matrix according to the simple indel coding method [50], as implemented in INDELCODER [51].

### Data analysis

#### Genetic diversity and differentiation

For each population, DNASP software [52–55] was employed to calculate the nucleotide diversity (*π*), the number of haplotypes, and the haplotype diversity (*H*_*d*_). The presence of phylogeographic structure was tested by using PERMUT [56–58] software (10,000 permutations) based on the parameter *N*_ST_ (a distance matrix based on the number of mutational steps between haplotypes) significantly higher than *G*_ST_ (haplotype frequencies). NETWORK software [59] was utilized to infer the relationship between the differentiation and evolution of haplotypes with the maximum parsimony median-joining model.

STRUCTURE software can detect the potential population structure more accurately duo to eliminating the interference of population locations [60–62]. With the associated gene frequency admixture model, for each *K* (*K* = 1–10), ten independent runs were conducted with an initial burn-in of 100,000 iterations and 1000,000 subsequent Markov Chain Monte Carlo (MCMC) steps. The log probability of data (*LnP*(*D*)) and the second rate of change in the likelihood distribution (Δ*K*) were applied to evaluate the potential clusters (*K*) [63]. The Q matrix was obtained by integrating the membership coefficient matrix of repeated operation from the results of STRUCTURE analysis by using CLUMPP software. And it was finally visualized as a diagram by using DISTRUCT software [60–63].

To quantify and assess the proportion of explainable genetic variance between groups and among populations within groups, an hierarchical analysis of molecular variance (AMOVA) [64] was conducted by ARLEQUIN software [54, 65], and then tested the significance with 1000 permutations.

#### Population demographic history

##### Mismatch distribution and neutrality tests

To infer whether there existed significant historic demographic expansion events in each geographical group, the mismatch distribution analysis was performed by ARLEQUIN software with 1000 parametric bootstraps [65, 66]. The parametric SSD (the sum of squared deviations) and HRag (Harpending’s raggedness index) were applied to appraise the goodness-of-fit under a sudden expansion model [67]. Meanwhile, the Tajima’s *D* (Tajima, 1989) [68] and Fu’s *F*_*S*_ (Fu, 1997) neutrality tests [69], as two alternate method, were used to infer potential expansions events.

The time of occurrence of expansion events was calculated by the following formula [66, 70]: *T* = *τ*/*2 u*, where *τ* was the parameter value for the mode of the mismatch distribution and *u* was the result of the formula *u* = *μkg*. The value *μ, k* and *g* represent the number of substitutions per site per year (s/s/y), the average sequence length under study, and the generation time in years, respectively [71, 72]. The parameter value α (maturity age of species, about 30 years) and [*S*/1 − *S*] (about 100; S, annual survival rate of adult individuals) was utilized to estimate the expansions time by using *g* = *a* + [*S*/(*1* − *S*)] [73, 74].

##### Divergence time

To relate the historic events and differentiation among *U. lamellosa* haplotypes, the divergence time was calculated using BEAST software with a calibrated molecular clock [44, 75–79]. The HKY, as the optimal model, was employed in BEAST analysis according to the Akaike information criterion (AIC) using jMODELTEST software [80, 81], and coalescence with a constant size was set as the prior tree. Due to the absence of records of ITS mutation rates in *U. lamellosa* or other Ulmaceae family members, the minimum and maximum values of all average ITS mutation rates of angiosperms [3.2 × 10^− 10^ and 9.0 × 10^− 9^ substitutions per site per year (s/s/y)] was selected to date the demographic events in this study [82].

The fossil records for *Ulmus* (15.97–13.82 Ma, https://www.paleobiodb.org/classic) were to be calibrated. The analysis was implemented for 50 million generations, with sampling frequency of once per 1000 generations. The log files were run in TRACER [83] and the 1000,000 generations were discarded as burn-in to ensure that the overall ESS value met the requirements (> 200). The TREEANNOTATOR software was applied to calculated an maximum clade credibility (MCC) tree with posterior probability (PP) limit > 0.5 using after removing the first 20% of trees as burn-in. And then the FIGTREE was used to visualize the final file [79, 84].

##### Approximate Bayesian computation

ABC analysis (Approximate Bayesian Computation) [76, 85–87] was performed in DIY-ABC software to model the potential demographic history and to evaluate the plausible evolutionary scenario of *U. lamellose* [88]. To simplify the number of model settings and operation time, we pooled all *U. lamellosa* populations into three geographical groups (THM, YSM, and YM). First, seven population divergence scenarios were tested with 7,000,000 coalescent simulations: scenario 1 (Simple split model), scenarios 2–4 (Hierarchical split model), and scenarios 5–7 (Isolation with admixture model). For the common parameters between models, the range was the same and the prior distribution was uniform. Based on the observation data closest to 1% of the simulated data, the posterior probability of each evolutionary event was predicted and the confidence interval up to 95% could be obtained by logistic regression. The optimal evolution scenario was filtered by using the highest a posteriori probability (PP) as the selection criterion.

In order to verify a goodness fit scenario, the “model checking” function in DIYABC software, namely principal component analysis (PCA), was used to analyze the simulated datasets (100,000) in the summary statistics. Based on the pseudo-observed datasets (PODs, 1000), type I and type II error rates was evaluated using the “evaluate confidence in scenario choice” functional option of DIYABC. According the above indices, the ancestral group of the optimal scenarios was further finally inferred.

##### Historical and contemporary gene flow

The coalescent method, implemented in MIGRATE-N software, was employed to investigate the historical gene flow pattern between the three geographical *U. lamellosa* groups [89–92]. The Bayesian method was used to estimate historical migration rates (*M* = *m*/*μ*, where *m* = migration rate) among groups and the effective population size (*θ* = 4*Neμ*, where *μ* = mutation rate) of different groups [89, 93]. The eq. 4*Nm* = *θ* ∗ *M* was employed to compute the number of migration per generation (*Nm*) of each population of *U. lamellosa* [89, 93, 94].

Furthermore, the gene flow analysis was performed under the HKY mutation model. It was run for five replications with constant mutation rates and starting parameters based on *F*_ST_ calculations. Each replicate was analyzed with 3 long chains and 10 short chains. The static heating treatment scheme corresponded to four temperature levels (1, 1.2, 1.5, and 3) and 1000,000 trees was discarded as burn-in. Then, the average of the results of five independent operations, namely the historical gene flow, would be obtained with a confidence interval of 95%.

BAYESASS software was used to calculate the contemporary gene flow [90, 92] between the three geographical *U. lamellosa* groups. This analysis was ran for 10,000,000 iterations with a sampling frequency of 1000 following a burn-in of 1000,000. The delta values for a (allele frequencies), m (migration), and f (inbreeding) was adjusted to 0.5 for guaranteeing the fixed numbers of changes were limited within the range from 20 to 60% of the total number of iterations. The data with the lowest deviance in 20 runs were selected in the further analysis. And the 95% confidence interval was calculated by mc ±1.96 × SD [74].

##### Isolation by distance and environment

To assess the influences of geographic isolation (IBD) and / or environmental conditions (IBE) on the observed genetic differentiation of *U. lamellosa* [54, 95, 96], the Mantel test of correlations was performed between genetic, geographic, and environmental distances, respectively.

Pairwise *F*_ST_ estimated from the ITS / *Aat* sequences was used as the genetic distances matrix. And the geographic distances between populations was calculated by using the GENALEX software [97]. While the matrix of the environmental distances was computed using PASSAGE software [98].

In addition, a multiple matrix regression with randomization (MMRR) was also performed to test whether the genetic distance responded to variations in the geographic and/or environmental distances via using the R package ‘PopGenReport’ [99, 100]. Correlation coefficients of the Mantel test (r) and regression coefficient of the MMRR (r^2^) and their significance were determined based on 9999 random permutations.

## Results

### Phylogeographical structures and genetic differentiation

The alignment of ITS and *Aat* sequences from 232 *U. lamellosa* individuals produced a consensus sequence of 652 and 392 base pairs, respectively. For the ITS sequences, eighteen haplotypes were defined with 27 polymorphic sites, including 17 parsimonious informative sites (Table 1). Between the 18 haplotypes, the most common was H1 (in 13 of 14 populations), followed by H2 (in 3 of 14 populations). Thirteen of the 14 populations were polymorphic, whereas only one population exhibited one haplotype (MS population, Table 1 and Fig. 1a). All haplotypes presented a ‘star-like’ network, where the common haplotype (H1) was in the central position and regarded as the most widespread one that connected other subordinate haplotypes (Fig. 1b).

**Table 1:** The estimated diversity indexes of *Ulmus lamellosa* populations

Groups	Populations	Sample sizes	ITS sequences			*Aat* sequences		
			Number haplotypes	Haplotype diversity (H_*d*_)	Nucleotide diversity (*π*)	Number haplotypes	Haplotype diversity (H_*d*_)	Nucleotide diversity (*π*)
THM	CC	17	H1(13), H6(2), H15(2)	0.396	0.00066	H2(3), H3(1), H4(5), H10(2), H15(4), H16(2)	0.857	0.00720
	HS	22	H1(20), H2(2)	0.318	0.00052	H12(11), H13(5), H14(6)	0.833	0.00808
	QLY	22	H1(18), H6(2), H7(2)	0.517	0.00140	H3(11), H4(6), H8(5)	0.833	0.00638
	SGS	15	H1(10), H2(5)	0.667	0.00103	H14(5), H19(5), H20(5)	1	0.01190
	MS	11	H1(11)	0	0	H17(11)	0	0
	JM	18	H1(12), H2(6)	0.667	0.00103	H10(18)	0	0
	HHG	15	H1(12), H2(3)	0.439	0.00073	H15(5), H21(5), H22(5)	1	0.01190
	LS	14	H17(7), H18(7)	1	0.00309	H17(7), H23(7)	1	0.00513
	TGS	14	H1(10), H8(4)	0.5	0.00155	H4(9), H9(5)	0.667	0.00680
	HLS	18	H1(10), H3(2), H4(2), H5(4)	0.636	0.00127	H1(5), H2(2), H3(6), H4(5)	0.81	0.00705
				0.359	0.00084		0.926	0.01506
YSM	SFS	20	H1(3), H8(2), H9(6), H10(7), H11(1), H12(1)	0.929	0.00839	H5(7), H6(7), H7(6)	0.8	0.00272
	WLS	18	H1(12), H16(6)	0.667	0.00309	H5(18)	0	0
				0.909	0.00939		0.714	0.00219
YM	JFS	12	H1(11), H5(1)	0.275	0.00063	H4(8), H18(4),	0.667	0.00680
	MLG	16	H1(12), H13(3), H14(1)	0.423	0.00109	H11(5), H12(5), H13(6)	1	0.00510
				0.332	0.00079		0.933	0.00969
Total		232	18	0.424	0.00212	23	0.941	0.01601

For the *Aat* data, twenty-three haplotypes (H1-H23) were defined, which contained 33 polymorphic sites, 26 of which were parsimonious informative sites (Table 1 and Fig. 1c). Among all of the haplotypes, the most common was H4 (in 5 of 14 populations). Eleven of the 14 populations possessed high haplotype diversity, and only one haplotype was found in the MS, LS, and WLS populations. As the most common and widespread haplotype, compared with the others s, H4 had more connections, was relatively situated in the middle of the network (Fig. 1d).

A high genetic diversity was detected for *U. lamellosa*. At the species level, the estimated haplotype diversity (H_*d* ITS_, H_*d Aat*_) was 0.424 and 0.941, whereas the nucleotide diversity (*π*_ITS_, *π*_*Aat*_) was 0.00212 and 0.01601, respectively. For the group level based on ITS data, the highest values of H_*d* ITS_ (0.909) and *π*_ITS_ (0.00939) existed in the YSM group, followed by the THM and YM groups. On the contrary, the H_*d Aat*_ and *π*_*Aat*_ values of the YSM group were slightly lower than that of the THM and YM groups via *Aat* data.

Between populations, the highest H_*d*_ value was detected in the different populations for different data (H_*d* ITS_ = 0.798 for the SFS population; H_*d Aat*_ = 1.000 for the SGS, HHG, SFS, and MLG populations). The highest *π*_ITS_ value also existed in the SFS population (0.00583), and the highest *π*_*Aat*_ value was found in the SGS and HHG populations (Table 1).

**Fig. 2:**
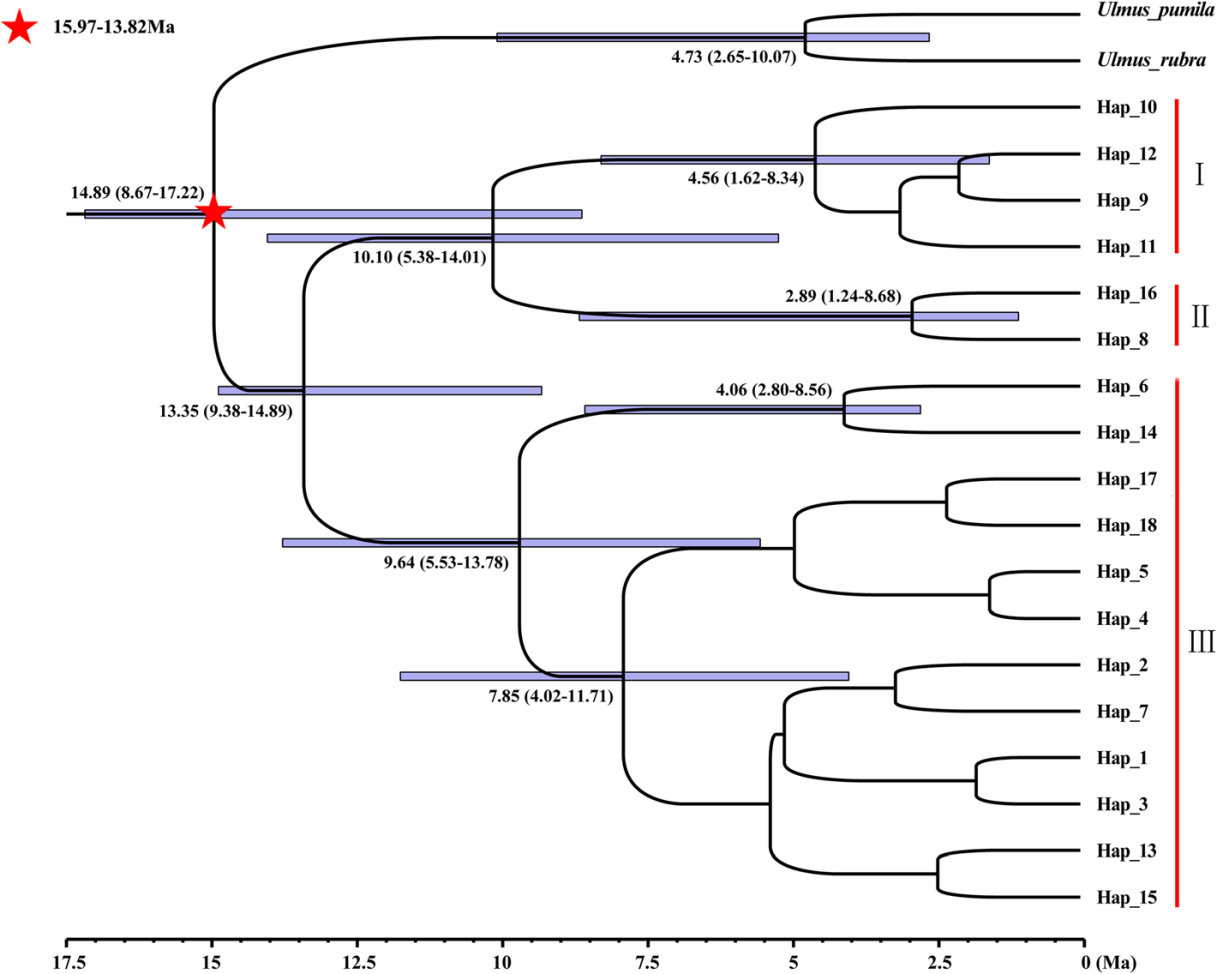
The chronogram for *Ulmus lamellosa* based on BEAST analysis of ITS sequences. Positions of the fossil calibrations were indicated by a five-pointed star. Divergence times were labeled on each node. Blue bars at nodes represented the 95% highest probability density (HPD) for the age of that node

The phylogenetic relationships of the ITS haplotypes (H1–H18) constructed using the Bayesian approach, revealed that all haplotypes were clustered into three major clades I-III (Fig. 2), which was essentially consistent with the geographical population distribution (namely YSM, THM, and YM groups). Clade I contained H9-H12, which were found in the YSM group. Clade II contained H8 and H16, which were found in THM and YSM groups. Clade III consisted of twelve haplotypes (H1–7, H13–15, H17, and H18) found in the THM and YM groups. For *Aat* data, 23 haplotypes were also clustered into three major clades (Additional file 2: Fig. S1), which was basically consistent with that of the haplotype network. Clade I and II contained most of the haplotypes, which were found in the THM and YSM groups. Clade III contained H5-H7, H15-H17, and H23, which were found in the THM and YM groups.

No matter the ITS or *Aat* data, the genetic differentiation between populations was significantly higher when computed using a distance matrix (*N*_ST ITS_ = 0.435; *N*_ST *Aat*_ = 0.738) than when using haplotype frequencies (*G*_ST ITS_ = 0.117; *G*_ST *Aat*_ = 0.192; *P* < 0.05), which indicated a significant phylogeographic structure within *U. lamellosa*.

For ITS, the STRUCTURE output showed that Δ*K* supported the existence of three clusters (Additional file 3: Fig. S2), which corresponded roughly with our geographic assignment to *U. lamellosa* (Table 1 and Fig. 1). Nevertheless, the STRUCTURE output for *Aat* data indicated that Δ*K* supported the existence of four clusters (Additional file 3: Fig. S2). However, when *K* = 4, the THM group was further subdivided into two clusters. Therefore, we preferred to divide the *U. lamellosa* into three clusters. Additionally, a higher inter-group differentiation of this species was confirmed by AMOVA analysis (*Φ*_ST ITS_ = 0.550, *Φ*_ST *Aat*_ = 0.674, *P* < 0.01, Table 2). Significant variation (ITS: 40.60%, *Aat*: 41.96%) occurred between the three groups (Table 2).

**Table 2:** Analysis of molecular variance (AMOVA) based on ITS and *Aat* sequences for *Ulmus lamellosa*

Source of variance	d.f^a^	SS^b^	Variance components	Variance percentage (%)	Fixation indices (*P* < 0.01)
Among groups	2	26.684 (39.147)	0.346 (0.981)	40.60 (41.96)	*Φ*_CT_ = 0.406(*Φ*_CT_ = 0.562)
Among populations within groups	11	15.287 (83.274)	0.123 (1.621)	14.43 (25.40)	*Φ*_SC_ = 0.243(*Φ*_SC_ = 0.254)
Within populations	231	43.631 (55.476)	0.383 (1.261)	44.97 (32.64)	*Φ*_ST_ = 0.550(*Φ*_ST_ = 0.674)

In parentheses--the results based on *Aat* data; Outside parentheses--the results based on ITS data

^a^, degrees of freedom; ^b^, sum of squares

### Inference of demographic history

#### Expansion events and divergence time

The SSD and HRag values were non-significant (*P* > 0.05) for all groups based on the ITS sequences. Nevertheless, the unimodal mismatch distribution for the THM group was a perfect fit to the expected expansion distribution (Table 3), as supported by a significant Tajima’s *D* statistic value. Thus, only the THM group of *U. lamellosa* experienced a recent demographic expansion, which occurred approximately 0.0038 Ma. For the *Aat* sequences, mismatch distributions were multimodal, and differed strongly from the predictions under a sudden population expansion model, which was evidenced by non-significant values (*P* > 0.01) of Tajima’s *D* statistics for the three groups (Table 3).

**Table 3:** Neutrality tests (Tajima’s *D* and Fu’s *F*_*S*_ tests) and mismatch distribution analyses

Groups	ITS sequences				*Aat* sequences			
	Neutrality tests		Mismatch distribution analyses		Neutrality tests		Mismatch distribution analyses	
	Tajima’s *D*	Fu’s *F*_*S*_	SSD (*P*_SSD_)	HRag (*P*_HRag_)	Tajima’s *D*	Fu’s *F*_*S*_	SSD (*P*_SSD_)	HRag (*P*_HRag_)
THM	−2.211 (*P* < 0.01)	−10.024 (*P* < 0.01)	0.00028 (0.800)	0.19533 (0.630)	−0.296 (*P* > 0.1)	−2.907 (*P* > 0.1)	0.010 (0.250)	0.013 (0.880)
YSM	0.498 (*P* > 0.1)	0.132 (*P* > 0.1)	0.404 (0.350)	0.072 (0.420)	0.413 (*P* > 0.1)	−0.071 (*P* > 0.1)	0.048 (0.170)	0.286 (0.240)
YM	−1.751 (*P* > 0.1)	−0.999 (*P* > 0.1)	0.009 (0.540)	0.234 (0.560)	1.394 (*P* > 0.1)	−0.839 (*P* > 0.1)	0.054 (0.350)	0.191 (0.330)
Total	−0.605 (*P* > 0.1)	−0.044 (N.A.)	0.069 (0.274)	0.336 (0.393)	0.015 (*P* > 0.1)	0.449 (N.A.)	0.105 (0.258)	0.307 (0.445)

When *Ulmus pumila* and *Ulmus rubra* were used as outgroups, the phylogenetic tree based on ITS data revealed that all of the haplotypes from *U. pumila* and *U. rubra* formed a monophyletic group, whereas the haplotypes of *U. lamellosa* formed another monophyletic group (Fig. 2). The haplotypes identified in the outgroups were divergent from all of the other haplotypes at ~ 14.89 Ma (95% HPD: 8.67–17.22 Ma). The eighteen haplotypes of *U. lamellosa* formed a lineage that was divergent at ~ 13.35 Ma (95% HPD: 9.38–14.89 Ma).

This suggested that the intraspecific divergence of all haplotypes most likely proceeded during the period between the Middle-Miocene to the early stage of the Late Miocene. Within *U. lamellosa*, all haplotypes could be roughly classified into three lineages, and the divergence times from clade I to clade III were 4.56 Ma (95% HPD: 1.62–8.34 Ma), 2.89 Ma (95% HPD: 1.24–8.68 Ma), and 9.64 Ma (95% HPD: 5.53–13.78 Ma), respectively.

#### ABC scenarios

**Fig. 3:**
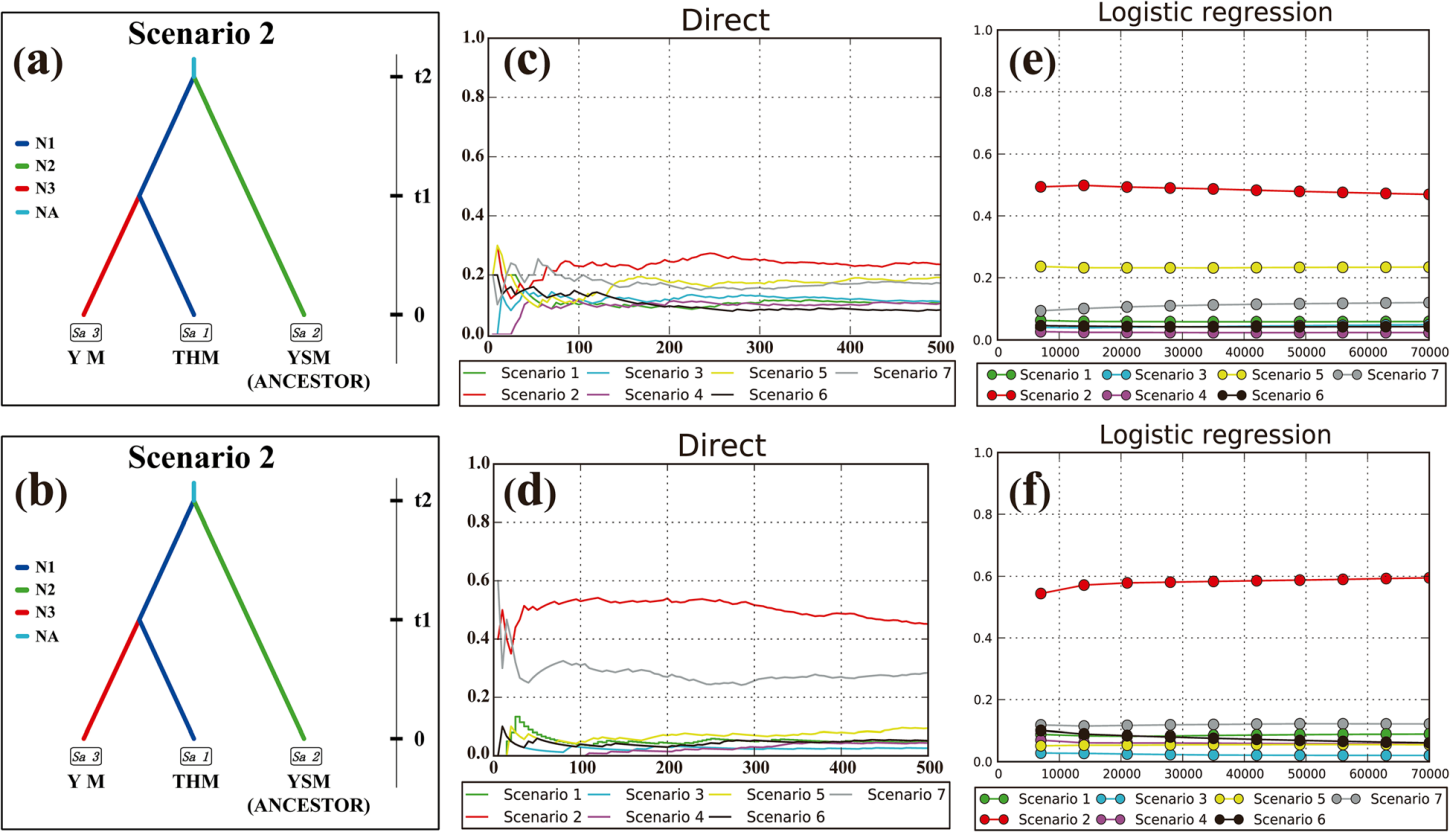
The Approximate Bayesian Computation analysis of *Ulmus lamellosa* assessed using DIYABC software. Time in generations was t (t2 ≥ t1). **a** optimal model (ITS), **c**, **e** posterior probabilities (ITS), **b** optimal model (*Aat*), **d**, **f** posterior probabilities (*Aat*)

An ABC framework permitted an evaluation for population divergence scenarios of *U. lamellosa.* Scenario 2 (Fig. 3a, b) had the highest posterior probability (ITS: Direct estimate: 0.4540, 95% CI: 0.0176–0.8904; Logistic regression: 0.5913, 95% CI: 0.5694–0.6132; *Aat*: Direct estimate: 0.2360, 95% CI: 0–0.6082; Logistic regression: 0.4695, 95% CI: 0.4584–0.4807) and did not overlap with other scenarios (Fig. 3c-f). This scenario was fitted to the observed data confirmed by PCA results. According to the model checking, the error for Scenario 2 was low (ITS: Direct approach: 0.061, Logistic approach: 0.075; *Aat*: Direct approach: 0.071, Logistic approach: 0.077). Consequently, Scenario 2, namely the YSM group, might be a common ancestor of the THM and YM groups, which diverged at time t2. Subsequently, the YM group diverged from the THM group at time t1 (Fig. 3a, b). Under Scenario 2, posterior media parameter estimates indicated that the first divergence occurred in 10.36 × 10^6^ (ITS: 95% CI: 4.23 × 10^6 _^ 12.88 × 10^6^), and 8.25 × 10^6^ (*Aat*: 95% CI: 2.56 × 10^6 _^ 12.73 × 10^6^) years with a generation time of 130 years.

The effective population size was estimated based on the sequencing data. For the ITS data, the THM group has the largest effective population size (5.92 × 10^5^, 95% CI: 1.88 × 10^5_^ 9.52× 10^5^), followed by the YSM group (4.56 × 10^5^, 95% CI: 1.06 × 10^5_^ 9.49 × 10^5^) and YM group (1.74 × 10^5^, 95%CI: 1.96 × 10^4_^ 8.59× 10^5^). For *Aat*, the median values of the effective population size were 2.35 × 10^5^ (95% CI: 4.65 × 10^4_^ 8.70× 10^5^) for the THM group, 2.79 × 10^5^ (95% CI: 4.50 × 10^4_^ 9.21 × 10^5^) for the YSM (the ancestral group), and 2.17 × 10^5^ (95% CI: 1.56 × 10^4_^ 9.27 × 10^5^) for the YM group. There was no significant difference in the effective population size between all groups based on *Aat* data.

#### Historical and contemporary gene flow

**Fig. 4:**
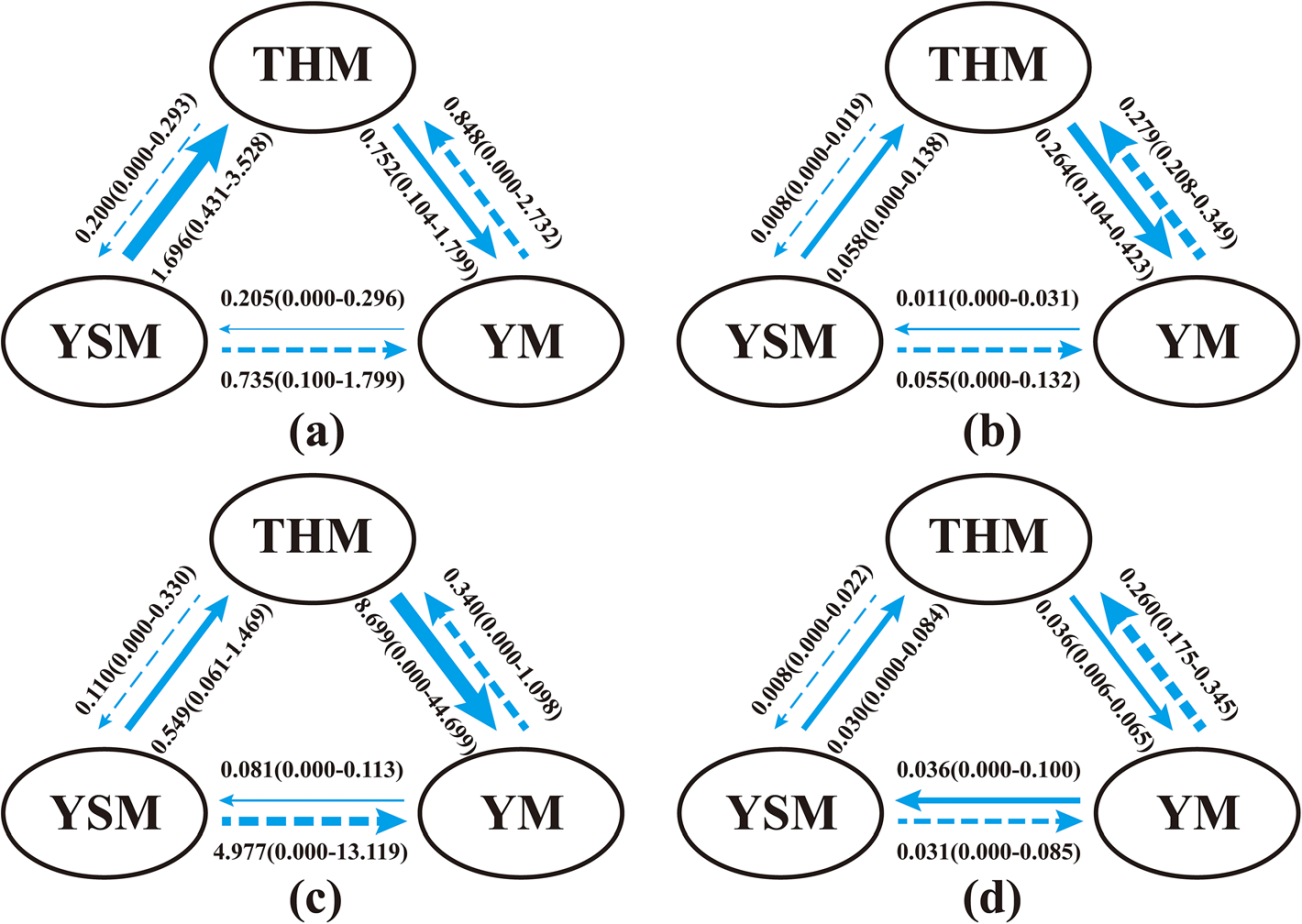
Estimates of historical gene flow using MIGRATE, contemporary gene flow using BEYASASS and 95% confidence intervals (CI) (in parentheses) within (**a**) historical gene flow based on ITS, (**b**) contemporary gene flow based on ITS, (**c**) historical gene flow based on *Aat*, and (**d**) contemporary gene flow based on *Aat*

The historical gene flow was asymmetric and higher than the modern gene flow (Fig. 4). For ITS sequences, the highest level of migration occurred from YSM to THM (1.696, 95% CI: 0.431–3.528), and the lowest level of gene flow occurred in the opposite direction (THM to YSM: 0.200, 95% CI: 0–0.293) (Fig. 4a). A higher level of migration occurred between THM and YM (YM to THM: 0.848, 95% CI: 0–2.732; THM to YM: 0.752, 95% CI: 0.104–1.799) (Fig. 4a) and the lower level occurred from YSM to YM (0.735, 95% CI: 0.100–1.799). According to the *Aat* sequences, the historical gene flow was generally high. A higher level of migration also occurred from THM to YM (8.699, 95% CI: 0–44.699) (Fig. 4c) than in the opposite direction. Nevertheless, a relatively high level of migration occurred from YSM to YM (4.977, 95% CI: 0–13.119).

The modern gene flow was low and asymmetrical, and BAYESASS analysis showed that the mean contemporary gene flow (m) based on ITS data between the three groups ranged from 0.008 to 0.279 (Fig. 4b). Similarly, a higher level of migration occurred from YSM-THM-YM (YSM-THM: 0.058, 95% CI: 0–0.138, THM-YM: 0.264, 95% CI: 0.104–0.423). However, a strong migration occurred from YM to THM (0.279, 95% CI: 0.208–0.349). For *Aat* sequences, the mean contemporary gene flow (m) between the three groups ranged from 0.008 to 0.260 (Fig. 4d). The highest level of migration occurred from YM to THM (0.260, 95% CI: 0.175–0.345). Further, a higher level of migration occurred from YSM-THM-YM (YSM-THM: 0.030, 95% CI: 0–0.084, THM-YM: 0.036, 95% CI: 0.006–0.065).

#### IBD and IBE

Pairwise *F*_ST_ values between populations (used as genetic distance) were correlated against the respective pairwise geographic and environmental distances (Additional file 4: Fig. S3). The positive correlation between the matrix of genetic distances and geographical distances was significant (ITS: r = 0.594, *P* < 0.05; *Aat*: r = 0.391, *P* < 0.05). However, the correlations between genetic distances estimated from the ITS /*Aat* and environmental distance were non-significant (*P* > 0.05).

Multiple matrix regression with randomization (MMRR) analyses also revealed a significant effect of geographical distance on the divergence of this species. Furthermore, IBD explained more genetic variation than IBE (ITS: MMRR ≈ 12.4 times as much; *Aat*: MMRR ≈ 3.7 times as much).

## Discussion

### Phylogeographic structures and genetic differentiation of U. lamellosa

A high level of genetic diversity was observed for *U. lamellosa* (H_*d* ITS_ = 0.424, *π*_ITS_ = 0.00212; H_*d Aat*_ = 0.941, *π*_*Aat*_ = 0.01601), which was similar with other plants, such as *Gentiana lawrencei* var. *farreri* (H_*d*_ = 0.414, *π* = 0.0026), and was consistent with the results of Liu et al. [40]. Compared with cpDNA data (H_*d*_ = 0.783, *π* = 0.00891) [40], the genetic diversity index of *U. lamellosa* was higher than that of ITS sequences and lower than that of *Aat* sequences (Table 1).

The different evolutionary trajectories and rates of nuclear and plastid markers might have been the cause of different degrees of variation, and reflected different biogeographic events such as pollen flow and seed dispersal [7, 16, 18, 44, 57, 101, 102]. High genetic diversity might reflect the accumulation of nucleotide mutations over long evolutionary *U. lamellosa* time-scales [103, 104]. In addition, *U. lamellosa* was widely distributed in Northern China (Additional file 1: Table S1 and Fig. 1). The geographic distribution of this species might also be one of the factors that determined high genetic diversity [105].

Being an ancestor of the other two groups (Fig. 3), the YSM group had a higher diversity and variation (Table 1), which suggested a smaller influence of historical climatic oscillations on this group. The Yanshan Mountains, which run roughly East-West, is an important mountain range in Northern China [106]. The heterogeneous geology of this mountain might have facilitated the existence of multiple glacial refugia for the plants [28, 107]. In our study, higher haplotype diversity was detected in the YSM group with five unique haplotypes (H8-H12, H16). Thus, the YSM group could be regarded as a refugia for *U. lamellosa* during the glacial period.

Meanwhile, QLY, SGS, JM, and HLS populations in the THM group (Table 1) exhibited the highest genetic diversity, although this group’s total genetic diversity was lower. It is possible that THM was another refugia for *U. lamellosa*. However, the YM group, having not only the lowest genetic diversity, but also being a descendent of THM and YSM (Fig. 3), was not regarded as a refugia in this study, which was inconsistent with Liu et al. [40].

A significant genetic differentiation between groups (Table 2, Fig. 1b, d, Additional file 2: Fig. S1) was revealed in the studied species, which suggested a long period of isolation during the distribution of *U. lamellosa*. Our results also found asymmetrical and lower contemporary gene flow (ITS: 0.0058–0.0690, *Aat*: 0.0083–0.1636, Fig. 4) among the three groups. Meanwhile, significant isolation by distance (r _ITS_ = 0.594, r _*Aat*_ = 0.39, *P* < 0.05; Additional file 4: Fig. S3) was presented in populations of this species. Such findings pinpointed that, as barriers, mountains might have driven population differentiation through long-term geographic isolation. More strikingly, most haplotypes were private as single groups. This pattern more likely resulted from long-term in situ diversification between isolated montane habitats.

Moreover, a strong phylogeographic structure was found, which was consistent with Liu et al. [40]. Theoretically, phylogeographic structures often arise as a consequence of restricted gene flow between populations and genetic drift within populations over relatively long timelines [56, 108, 109]. With the uplift of mountains in Northern China, the *U. lamellosa* habitats were naturally fragmented [38]. Climatic fluctuations that occurred during inter−/postglacial would further accelerate the habitat fragmentation of this species.

Such isolation made it difficult for pollinators to locate or access flowers and thus formed potential barriers to gene flow. Further, due to natural decomposition and animal consumption, the dispersal of *U. lamellosa* seeds was also restricted [36, 57]. The historical and modern restricted gene flow (Fig. 4) between groups supported this view. In addition, fragmentation is a consequence of isolation that can arise either from geographic or environmental factors [40]. The fragmented habitats of *U. lamellosa* could not counteract the effects of genetic drift. Therefore, limited gene flow and genetic drift were the primary causes of the phylogeographical structure for *U. lamellosa*.

### Demographic population history of U. lamellosa

Neither ITS nor *Aat* sequences indicated that most of the *U. lamellosa* populations did not experience expansion, which was supported by the mismatched distribution (Table 3), as well as non-significance Tajima’s *D* and Fu’s *F*_*S*_ values, and consistent with Liu et al. [40]. Interestingly, only the THM group (ITS data showed) of *U. lamellosa* experienced a recent demographic expansion that occurred ~ 3800 years ago. This was strongly supported by ABC analyses, which revealed that the THM group had the larger effective population size (5.92 × 10^5^, 95% CI: 1.88 × 10^5_^ 9.52× 10^5^) over ancestral populations (YSM group). Furthermore, a higher gene flow occurred from THM to YM (Fig. 4).

Despite the absence of major Quaternary glaciations, significant climatic oscillations still occurred in Northern China [110–112]. During this period, plant communities might have experienced the upward and downward migration along the local altitude, rather than southward retreat or northward advance in latitude [113]. Therefore, *U. lamellosa* might have taken refuge in situ and experienced only limited expansion during the interglacial period. The geographical distribution of haplotypes also supported this expansion pattern in the historical demography of *U. lamellosa*, namely the occurrence of common haplotypes H1 (ITS data) and H4 (*Aat* data) in the populations (Fig. 1a, c).

The intraspecific divergence of all *U. lamellosa* ITS haplotypes (13.35 Ma, 95% HPD: 9.38–14.89 Ma) likely began during the period between Middle-Miocene and the early stage of the Late Miocene (Fig. 2). This estimated divergence time was earlier than the Late Miocene based on cpDNA data (9.27 Ma, 95% HPD: 5.17–13.33 Ma) [40]. The Miocene to Pliocene period is an important differentiation epoch for this tree species in China. The Miocene was a period of climatic instability and abrupt environmental change. A global cooling event reoccurred in the Miocene (14 Ma) [11, 14, 114]. Prior to and following 16 ~ 12 Ma, the monsoon circulation was significantly enhanced in Asia [14, 115–117]. Most areas of Northern China were gradually altered from the arid climate, which was controlled by the original planetary climate, to the present warm temperate monsoon climate system [118, 119]. Thus, Northern China was not only under the control of global climate change, but also affected by the expansion of an arid climate from the West to East [120]. Paleoclimate oscillation caused by corresponding events prompted the *U. lamellosa* populations to migrate from the Yanshan Mountains (YSM, ancestral group) to the Taihang Mountains (THM group, the descendent).

Furthermore, the deformation of the Taihang Mountains occurred with the significant uplift of the Tibetan-Himalayan Plateau [14, 121–123]. The uplift height of the northern section of the Taihang Mountains was only half of the Western portion of the Yanshan Mountains during the Miocene [121]. The low height of the Taihang Mountains did not develop as a geographical barrier and did not block *U. lamellosa* migration, from the northwest and northeast, which is supported by the more frequent gene exchange between the three groups (Fig. 4 and Additional file 3: Fig. S2).

In ABC, the divergence between the THM and YM groups was dated back to from 3.54 Ma (ITS) and 3.25 Ma (*Aat*) (ITS: 95% CI: 5.36 × 10^5^–6.24× 10^6^; *Aat*: 95% CI: 3.82 × 10^5^–6.28 × 10^6)^ (Fig. 3), which corresponds to the Pliocene. The YM group diverged from the THM group (Fig. 3a, b), was a descendant of THM. During the Pliocene, most areas of the Taihang Mountains had only an < 800 m elevation [121].

From the Eocene to the end of the Pliocene, the Yinshan Mountains emerged rapidly, which might have hindered glacial activities and climatic oscillations. Consequently, the *U. lamellosa* populations of the Taihang Mountains began to migrate northward to form the YM group. The higher gene flow from the THM to YM group (Fig. 4) supported the above points.

Certainly, the demographic history of *U. lamellosa* had also been influenced by climatic oscillations and historical processes during the Quaternary [14, 124], which was supported by the divergence time of Clade II (2.89 Ma, 95% HPD: 1.24–8.86) (Fig. 2). During this period, the Taihang and Yanshan Mountains were uplifted sharply along with the Himalayan orogeny movement to finally form in the Pleistocene (~ 2.5 Ma B.P.) [16]. Meanwhile, repeated glacial periods had occurred since the Quaternary. These events would further promote differentiation between *U. lamellosa* populations and lead to the current pattern of this species*.*

## Conclusions

Higher genetic diversity and significant genetic variations occurred in *U. lamellosa* populations. During glaciation, the regions of Yanshan and Taihang Mountains might be regarded as refugia for this long-lived tree species. The divergence of *U. lamellosa* intraspecies haplotypes occurred during the Miocene-Pliocene, which was associated with major Tertiary geological and/or climatic events in Northern China. Subsequently, the series of Mountains (e.g., Yanshan and Taihang Mountains) uplifted in Northern China once Tertiary and Quaternary climatic oscillations blocked gene exchange and further promoted the divergence of populations.

In summary, the causes of the phylogeography pattern of *U. lamellosa* were suggested based on geological evidence and paleoclimate data. The results in this study may provide insights into how the endangered *U. lamellosa* species might be protected and utilizing. Further, they may play an important role in the exploration of genetic differentiation patterns and dynamic changes of other tree species, while offering a scientific basis toward understanding the evolutionary history and ecological adaption of forests in Northern China.

## Supplementary Information

**Additional file 1: Table S1.** Locations and sizes of individuals in the sampled populations of *Ulmus lamellose*.

**Additional file 2: Figure S1.** The maximum likelihood tree of single-copy nuclear gene *Aat* haplotypes.

**Additional file 3: Figure S2.** Results of Bayesian clustering analysis conducted by STRUCTURE. The clustering patterns of ITS (a) and *Aat* (b) by three clusters (*K* = 3). The clustering patterns of *Aat* (c) by four clusters (*K* = 4).

**Additional file 4: Figure S3.** Correlation between geographic distance and pairwise *F*_ST_/environmental distance for *Ulmus lamellosa*. (a), (b): relationship estimated by ITS and (c), (d): relationship estimated by *Aat*.

## Data Availability

All sequencing data generated or analyzed during this study has been submitted to NCBI GENEBANK database (https://www.ncbi.nlm.nih.gov/genbank/). The accession numbers were MW091502 - MW091519 for the nuclear ribosomal internal transcribed spacer regions (ITS) analysis data. And the single-copy gene (*Aat*) data accession numbers were MW116046 - MW116068. The datasets used and/or analyzed during the current study are also available from the corresponding author (Yiling Wang, ylwangbj@hotmail.com) on reasonable request.
