# Supplementary figures and images for: Demographic history and genetic differentiation of an endemic and endangered Ulmus lamellosa (Ulmus)
Source: BMC Plant Biol. 2020 Nov 17;20:526. doi: 10.1186/s12870-020-02723-7 (PMC7672979; doi:10.1186/s12870-020-02723-7)

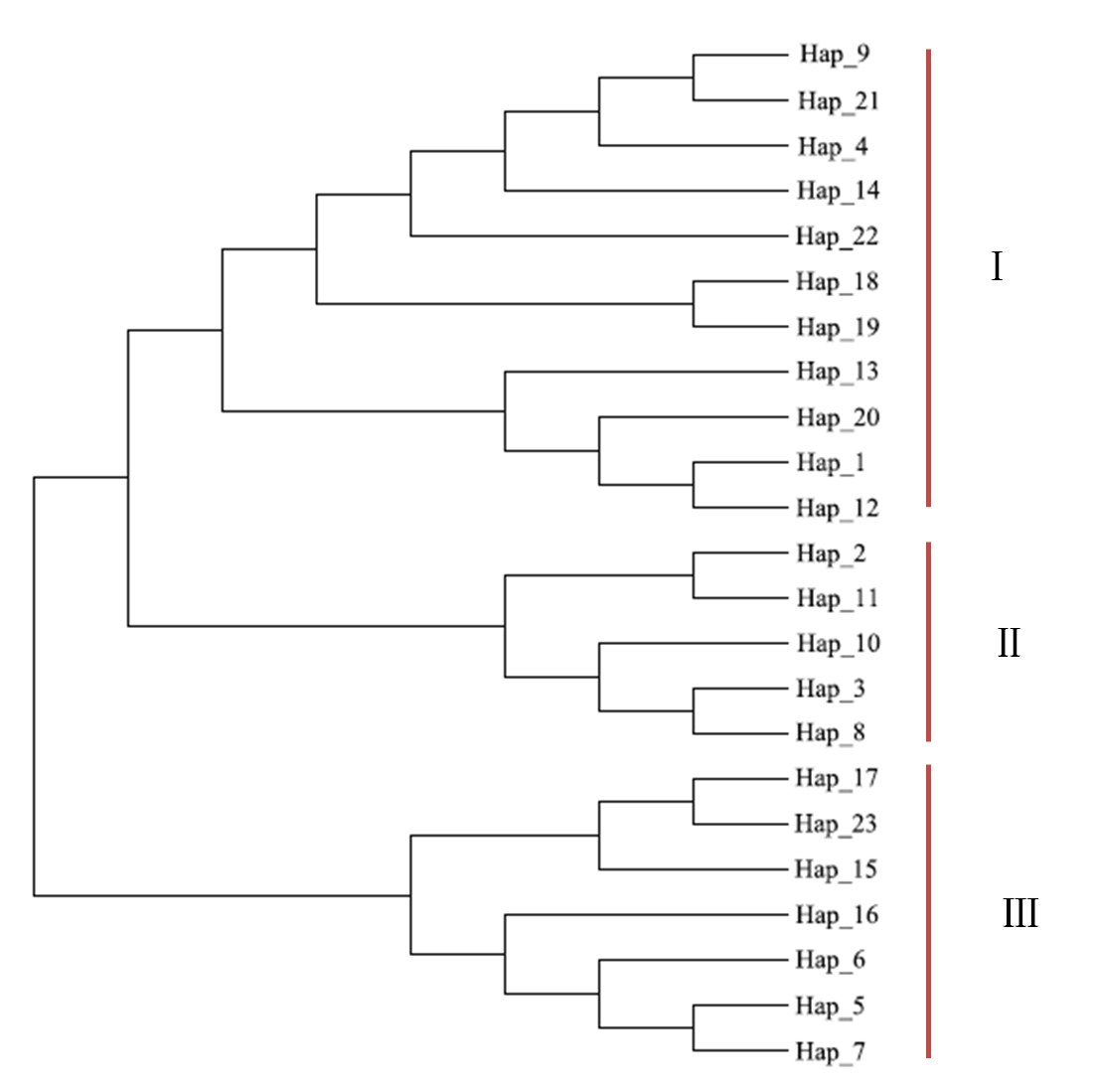


Fig. S1. The maximum likelihood tree of single-copy nuclear gene *Aat* haplotypes

Supplement: Supplementary file 2 — Additional file 2: Figure S1. The maximum likelihood tree of single-copy nuclear gene Aat haplotypes. [file 12870_2020_2723_MOESM2_ESM.doc]
