# Supplementary material for: Demographic history and genetic differentiation of an endemic and endangered Ulmus lamellosa (Ulmus)
Source: BMC Plant Biol. 2020 Nov 17;20:526. doi: 10.1186/s12870-020-02723-7 (PMC7672979; doi:10.1186/s12870-020-02723-7)
Supplement: Supplementary file 3 — Additional file 3: Figure S2. Results of Bayesian clustering analysis conducted by STRUCTURE. The clustering patterns of ITS (a) and Aat (b) by three clusters (K = 3). The clustering patterns of Aat (c) by four clusters (K = 4). [file 12870_2020_2723_MOESM3_ESM.doc]

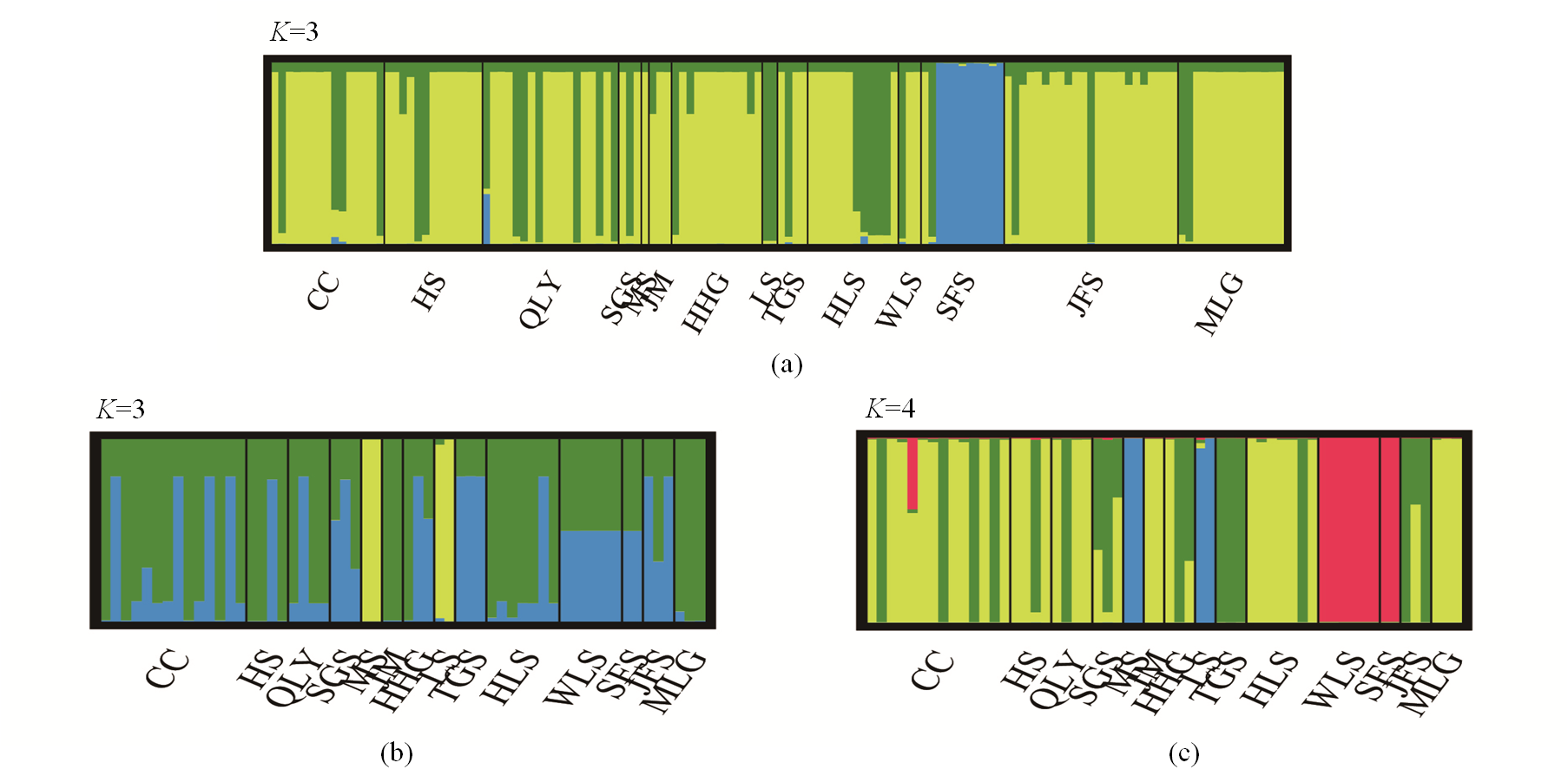


Fig. S2. Results of Bayesian clustering analysis conducted by STRUCTURE. The clustering patterns of ITS (a) and *Aat* (b) by three clusters (*K* = 3). The clustering patterns of *Aat* (c) by four clusters (*K* = 4).
