# Supplementary material for: Demographic history and genetic differentiation of an endemic and endangered Ulmus lamellosa (Ulmus)
Source: BMC Plant Biol. 2020 Nov 17;20:526. doi: 10.1186/s12870-020-02723-7 (PMC7672979; doi:10.1186/s12870-020-02723-7)
Supplement: Supplementary file 4 — Additional file 4: Figure S3. Correlation between geographic distance and pairwise FST/environmental distance for Ulmus lamellosa. (a), (b): relationship estimated by ITS and (c), (d): relationship estimated by Aat. [file 12870_2020_2723_MOESM4_ESM.doc]

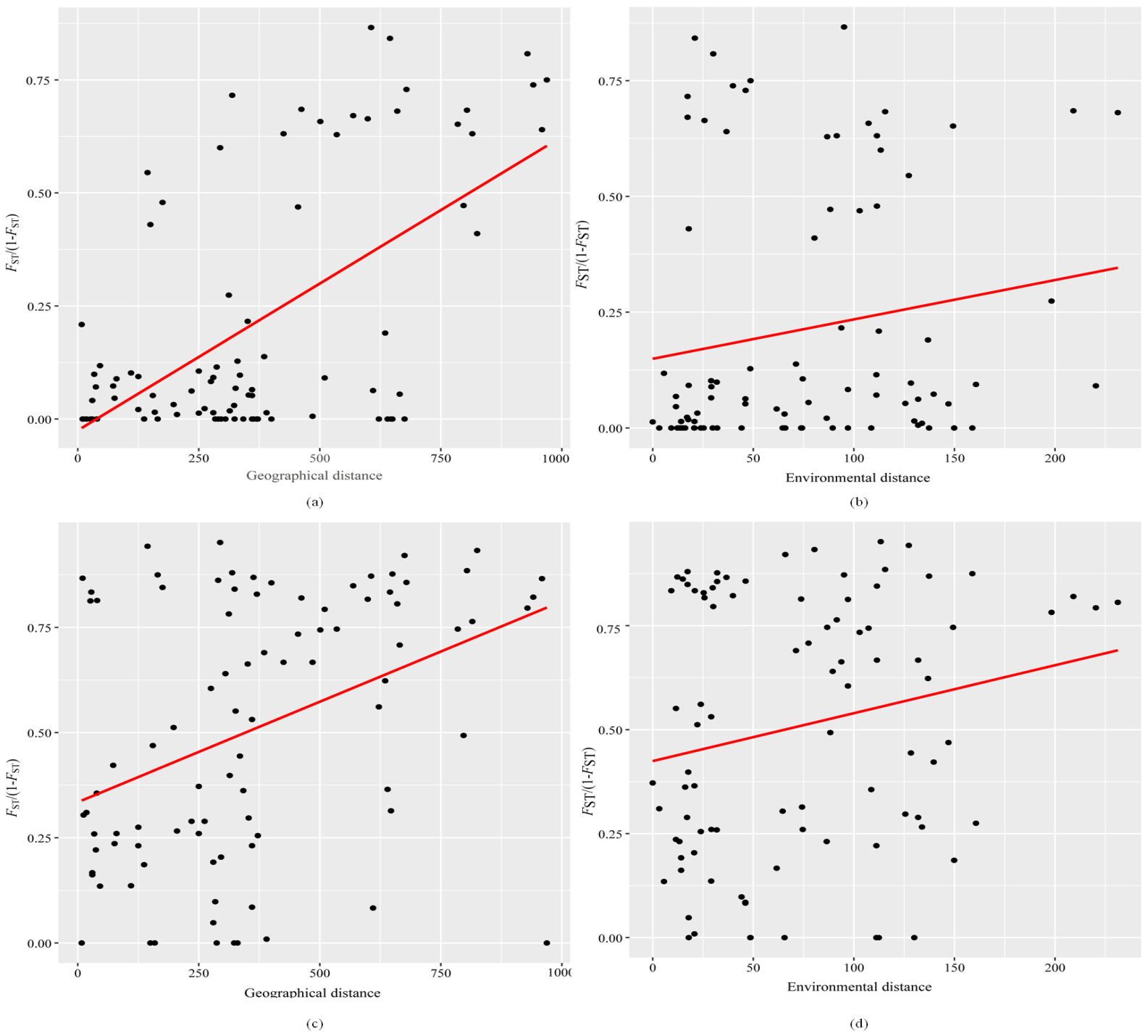


Fig. S3. Correlation between geographic distance and pairwise *F*_ST_/environmental distance for *Ulmus lamellosa.* (a), (b): relationship estimated by ITS and (c), (d): relationship estimated by *Aat*.
